# 

**Published:** 1899-05

**Authors:** 


### CONTENTS:

### SELECTIONS.

Article I.—A Few Considerations in Filling Teeth. C. N. Johnson, L. D. S., D. D. S ...... 1
" II.—On Restoring Consciousness. Dr. Bowles ...... 12
" III.—Porcelain Inlays. H. S. Guilford, D. D. S., Ph. D .... 13
" IV.—Some Notes on the Mixing of Amalgams. T. H. Dodd. 17
" V.—Arsenious Treatment for the Removal of Tumors from the Mouth. Dr. J. L. Mewborn ...... 19
" VI.—"Toothache." D. D. Atkinson. D. D. S ...... 22
" VII.—Removable vs Fixed Bridge Work. T. E. Turner, D. D. S ...... 24
" VIII.—Dentists in the Army. Geo. W. Griswold, D. D S... 30
" IX.—Pyorrhœa Alveolaris.—Personal Experence in Treatment. W. G. Beers, L D. S., D. S ...... 31
" X.—Silver Nitrate in the Treatment of Pulpless Teeth. Dr. L. G. Noel ...... 35
" XI.—Opportunities for Success. J. H. Grant, D. D. S ...... 38
" XII.—Surgical Hints ...... 39
" XIII.—The Influence of Pregnancy on the Caries of the Teeth. Dr. S. Bird ...... 41

### MONTHLY SUMMARY.

Broke a Tooth and Bled to Death ...... 42

Fatalities From Anesthetics ...... 44

Pyorrhœa Alveolaris Treatment ...... 44

A Rubber Substitute From Corn ...... 45

Sensitive Dentine ...... 45

Eucaine ...... 45

Repairing a Gold Crown ...... 46

Smokers' Teeth ...... 46

Where Pyrozone is Effective ...... 46

Asepsis ...... 47

Filling Frail Teeth ...... 47

Suggestions on Use of the Hypodermic Syringe ...... 48

A Low Fusible Metal ...... 48

Dental Fees ...... 48

